# Diagnostic Characteristics of Serological-Based COVID-19 Testing: A Systematic Review and Meta-Analysis

**DOI:** 10.6061/clinics/2020/e2212

**Published:** 2020-08-06

**Authors:** Diogo Turiani Hourneaux de Moura, Thomas R. McCarty, Igor Braga Ribeiro, Mateus Pereira Funari, Pedro Victor Aniz Gomes de Oliveira, Antonio Afonso de Miranda Neto, Epifânio Silvino do Monte Júnior, Francisco Tustumi, Wanderley Marques Bernardo, Eduardo Guimarães Hourneaux de Moura, Christopher C. Thompson

**Affiliations:** I Hospital das Clinicas HCFMUSP, Faculdade de Medicina, Universidade de Sao Paulo, Sao Paulo, SP, BR.; II Brigham and Women’s Hospital, Harvard Medical School, Boston, MA, United States.

**Keywords:** COVID-19, Coronavirus, SARS-CoV- 2, Serological, Diagnosis

## Abstract

Serologic testing for severe acute respiratory syndrome coronavirus 2 (SARS-CoV-2) promises to assist in assessing exposure to and confirming the diagnosis of coronavirus disease 2019 (COVID-19), and to provide a roadmap for reopening countries worldwide. Considering this, a proper understanding of serologic-based diagnostic testing characteristics is critical. The aim of this study was to perform a structured systematic review and meta-analysis to evaluate the diagnostic characteristics of serological-based COVID-19 testing. Electronic searches were performed using Medline (PubMed), EMBASE, and Cochrane Library. Full-text observational studies that reported IgG or IgM diagnostic yield and used nucleic acid amplification tests (NAATs) of respiratory tract specimens, as a the reference standard in English language were included. A bivariate model was used to compute pooled sensitivity, specificity, positive/negative likelihood ratio (LR), diagnostic odds ratio (OR), and summary receiver operating characteristic curve (SROC) with corresponding 95% confidence intervals (CIs). Five studies (n=1,166 individual tests) met inclusion criteria. The pooled sensitivity, specificity, and diagnostic accuracy for IgG was 81% [(95% CI, 61-92);I^2^=95.28], 97% [(95% CI, 78-100);I^2^=97.80], and 93% (95% CI, 91-95), respectively. The sensitivity, specificity, and accuracy for IgM antibodies was 80% [(95% CI, 57-92);I^2^=94.63], 96% [(95% CI, 81-99);I^2^=92.96] and 95% (95% CI, 92-96). This meta-analysis demonstrates suboptimal sensitivity and specificity of serologic-based diagnostic testing for SARS-CoV-2 and suggests that antibody testing alone, in its current form, is unlikely to be an adequate solution to the difficulties posed by COVID-19 and in guiding future policy decisions regarding social distancing and reopening of the economy worldwide.

## INTRODUCTION

In late November 2019, an outbreak of viral respiratory illness in Wuhan, China, attracted worldwide attention. This severe acute respiratory syndrome coronavirus 2 (SARS-CoV-2) infection (1), which was later identified as the novel coronavirus disease 2019 (COVID-19), has since rapidly spread across the globe infecting more than 3.7 million individuals and resulting in approximately 260,000 deaths at the writing of this manuscript (2,3). Declared a pandemic by the World Health Organization (WHO), the virus has continued to spread across the globe despite public health responses aimed at containing the disease (4-7). Early implementation of social distancing and other public health mitigation strategies have been shown to reduce the number of hospitalizations and deaths; however, transmission of SARS-CoV-2 among asymptomatic individuals and those with minimal symptoms early in the course of infection argue for large-scale testing, rapid diagnosis, standardized practices for isolation, and rigorous case tracking (8-11). Test-based strategies may also allow for less austere social distancing measures and provide an alternative that is less destructive to the global economy (12).

Though massive testing efforts are a cornerstone of strategies aimed to reduce the social and economic burden of COVID-19, the accuracy of commercially available tests for COVID-19 remain unclear. Currently nucleic acid amplification tests (NAATs), such as real-time reverse-transcription polymerase chain reaction (real-time RT-PCR) remains the primary method for diagnosis of COVID-19 (13). This RT-PCR technique involves the reverse transcription of SARS-CoV-2 RNA into complementary DNA (cDNA) strands, followed by amplification of specific regions of the cDNA to identify the virus (14,15). However, these tests have several disadvantages including, prolonged sample processing times, need for specialized equipment and reagents, as well as a reliance on appropriate swab technique. Additionally, NAAT tests have shown an unnegligibler false-negative rate in the diagnosis of suspected cases. These limitations pose a threat to the community and complicate development of epidemic prevention policies (16-20). While this test is still the most effective method to date for the diagnosis of active COVID-19, serologic-based antibody testing to assist with known exposure to SARS-CoV-2 remains pivotal to accurately assessing the burden of disease.

Serologic-based tests such as enzyme-linked immunoassay (ELISA) for specific IgM and IgG antibodies are needed to diagnose the general population and may serve as a roadmap to reopen the global economy. Detection of IgM antibodies are often interpreted as an indicator of acute infection while the detection of IgG antibodies represents previous infection/immunity (18,21,22). This testing strategy would require a high test sensitivity and specificity, aimed at minimizing false negative and positive results. Regarding public policies, IgM and IgG tests also have the advantage of providing faster results compared to NAAT (15,23-26). Given the possible benefits of serologic-based IgM and IgG testing, and the importance to management of the current pandemic, proper understanding and assessment of these diagnostic tests is critical. Therefore, we aim to perform a structured systematic review and meta-analysis to evaluate the diagnostic characteristics of serological-based testing (IgG and IgM) for COVID-19.

## METHODS

### Protocol and Registration

This systematic review and meta-analysis was conducted following the Preferred Reporting Items for Systematic Reviews and Meta-Analyses (PRISMA) recommendations (27) (Appendix - Supplementary File 1). The review was registered in PROSPERO international database (28) (CRD42020182315).

### Literature Search Strategy

A literature search was performed for eletronic databases including Medline (PubMed), EMBASE, Cochrane, LILACS, Scopus, and CINAHL through 02 May, 2020. Individualized literature search strategies were developed to identify full-text manuscripts using the following search strategy for all databases: (COVID-19 OR coronovirus OR SARS-CoV-2 OR Human coronavirus OR 2019-nCOV). After duplicate articles were removed, the titles and abstracts of potential studies were screened for eligibility. We also searched relevant websites (29-31). The reference lists of studies of interest were then manually reviewed for additional articles by cross checking bibliographies. Two reviewers independently screened the titles and abstracts of all the articles according to the inclusion and exclusion criteria. Disagreements between the reviewers were resolved via a discussion with all authors.

### Eligibility Criteria

Observational studies including two arms (index test and reference standard) were evaluated. Patients with serologic-based testing such as IgM and/or IgG) and NAAT, such as rRT-PCR, which is the reference standard method were included. Studies were only included for testing among adult patients (age >18 years) and if data was available for the construction of two-by-two contingency tables. The number of true positives (TP), true negatives (TN), false positives (FP), and false negatives (FN) were then abstracted from full-text manuscripts. Only full-text, English language manuscripts were assessed. Case reports, editorials, systematic reviews, and non-sequential case series were not eligible for inclusion.

### Outcome Measures and Data Extraction

The main outcome of this systematic review and meta-analysis was the accuracy of serologic-based tests for the diagnosis of COVID-19. Secondary outcome measures included sensitivity, specificity, positive predictive value (PPV), negative predictive value (NPV), positive likelihood ratio (+LR), negative likelihood ratio (-LR), and diagnostic odds ratio (OR). The reference standard, which served as control arm to compare serologic-based IgG and IgM tests was NAAT testing. Then, using these data, a definition of true disease and non-disease cases was obtained. Data extracted from the literature search also included study characteristics, number of included patients, the reference standard and data regarding the TP, FP, FN, and TN values.

### Assessment of Clinical Utility

In an effort to determine clinical utility of IgG and IgM serologic-based testing for COVID-19, a probability modifying plot and Fagan nomogram were constructed. The Fagan nomogram is a graphical tool for estimating how much the result of a diagnostic test changes the probability that a patient has a disease. Additionally, a probability modifying plot was created as a graphical sensitivity analysis of predictive value across a continuum (i.e., low to high prevalence defining low to high-risk populations) (32,33).

### Assessment of Methodologic Quality

To evaluate the methodologic quality of individual studies, quality assessment of diagnostic accuracy studies (QUADAS-2) was performed (34). This is an evidence-based tool for assessment of quality in systematic reviews of diagnostic accuracy studies with each key domain using a set of signaling questions to assess bias and applicability. Only studies that provided all information necessary to complete the table for at least one analysis were included in the meta-analysis. This process was performed by three independent reviewers and disagreements were resolved by consensus among all authors.

### Investigation of Heterogeneity

Heterogeneity is generally accepted to be present within diagnostic test accuracy reviews (35). As such, random effects models were fitted by default. Heterogeneity was assessed for the individual meta-analyses using the I^2^ statistic with significant heterogeneity defined as I^2^ >50%. Further quantification of heterogeneity was categorized based upon I^2^ with values of 25%, 50%, and 75% indicating low, moderate, and high amounts of heterogeneity, respectively.

### Statistical Analysis

This systematic review and meta-analysis was performed according to the Cochrane Diagnostic Test Accuracy working group methodology. Two-by-two contingency tables were conducted for testing characteristics of serologic-based testing (IgM and IgG) as well as NAAT testing to calculate diagnostic performance (sensitivity, specificity, +/-LR, diagnostic odds ratio, and diagnostic accuracy) for the diagnosis of COVID. Data on test accuracy and disease prevalence as well as TP, TN, FP, and FN allowed for calculation of diagnostic performance with measures of statistical uncertainty (e.g. 95% confidence intervals). The confidence intervals (CIs) were calculated using the F distribution method. For 0 values, 0.5 was added, as described by Cox and Snell (36).

A bivariate model was used to compute combined weighted sensitivity, specificity, +LR, -LR, diagnostic OR, and the summary receiver operating characteristic curves (SROC) with corresponding 95% CI. The SROC curves were created using the Moses-Littenberg linear model. Based upon the SROC, the area under the curve (AUC) was used to determine diagnostic accuracy. If a SROC could not be constructed, the accuracy was calculated manually from non-pooled provided sensitivity and specificity. A random effects model was utilized based upon heterogeneity inherent to diagnostic accuracy meta-analyses. Diagnostic performance was analyzed using the STATA 15.0 software package (Stata Corp LP, College Station, TX, USA) with midas user-written command.

## RESULTS

### Literature Search Results and Study Characteristics

**Figure 1:**
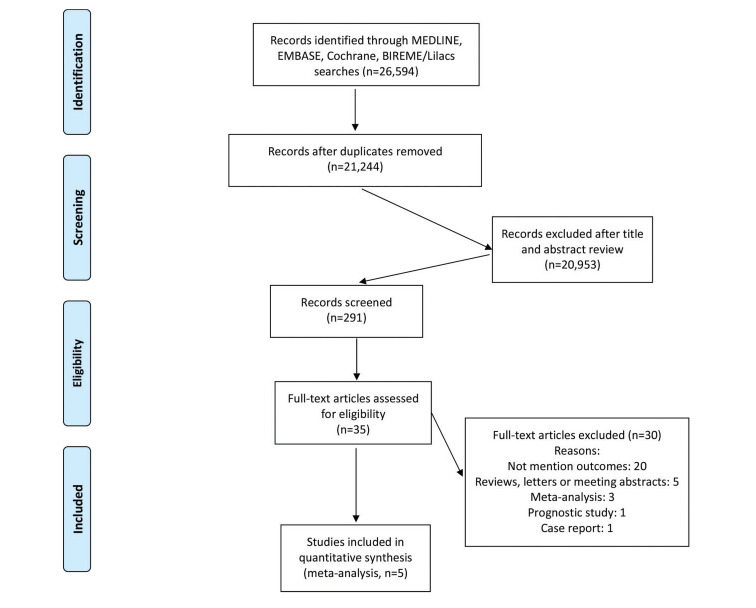
Prisma Flow Chart.

A total of 26,594 studies were originally extracted based upon our previously described literature search methodology. From these, 21,244 studies were removed due to duplicate records and 291 studies were excluded after title and abstract screening. The 35 remaining studies were then evaluated using the pre-specified inclusion and exclusion criteria (Figure 1). This resulted in a total of 5 studies (22,37-40) including 1,166 patients undergoing serologic-based testing for COVID-19 evaluation. The characteristics of the included studies, along with individual study results, are shown in Table 1.

### Risk of bias

The quality of the included studies was evaluated according to the QUADAS-2. Risk of bias and applicability concerns of the 5 studies are shown in Appendix - Supplementary File 2. The quality of the included studies was considered adequate. The risk of bias regarding patient selection was unclear. On the other domains, a low risk of bias was perceived. There was low concern for applicability regarding the first three QUADAS-2 domains for all included studies.

### Diagnostic Testing Characteristics

A total of 1,166 patients underwent serological testing for COVID-19. Of these, 623 had the disease. Therefore, the prevalence of COVID-19 in the studied population was 53.43%.

**Figure 2:**
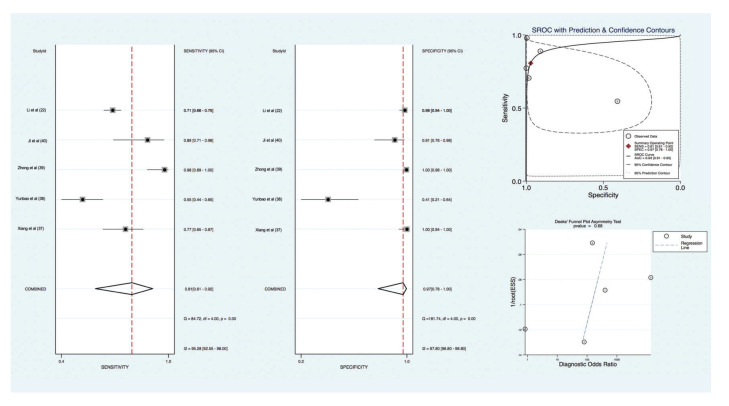
IgG test pooled diagnostic value for diagnosis of SARS-CoV-2 infection.

With regards to the diagnosis of previous SARS-CoV-2 infection based upon ELISA serum testing, compared to the reference standard (RT-PCR), the pooled sensitivity and specificity for IgG was 81% [(95% CI, 61 to 92); I^2^=95.28]. and 97% [(95% CI, 78 to 100); I^2^=97.80], respectively. The +LR for IgG testing was 28.63 [(95% CI, 2.88 to 284.69); I^2^=97.60] with a -LR of 0.20 [(95% CI, 0.09 to 0.45); I^2^=94.84]. The diagnostic OR was 144 (95% CI, 8 to 2650). Diagnostic accuracy as measured by SROC demonstrated an accuracy of 93% (95% CI, 91 to 95). There was no evidence of publication bias based upon a Deeks’ funnel plot (*p*=0.68) (Figure 2).

**Figure 3:**
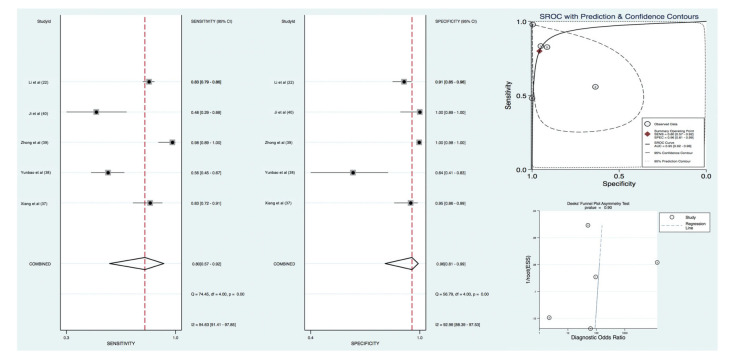
IgM test pooled diagnostic value for diagnosis of SARS-CoV-2 infection.

For acute or active COVID-19, the pooled sensitivity and specificity for IgM antibodies was 80% [(95% CI, 57 to 92); I^2^=94.63]. and 96% [(95% CI, 81 to 99); I^2^=92.96], respectively. The +LR and -LR for IgM-based serologic testing was 18.8 [(95% CI, 3.4 to 105.7); I^2^=90.14] and 0.21 [(95% CI, 0.08 to 0.53); I^2^=95.11]. The diagnostic OR was 90 (95% CI, 8 to 1058). Diagnostic accuracy was 95% (95% CI, 92 to 96). There was no evidence of publication bias based upon a Deeks’ funnel plot (*p*=0.90) (Figure 3).

### Clinical Utility

**Figure 4:**
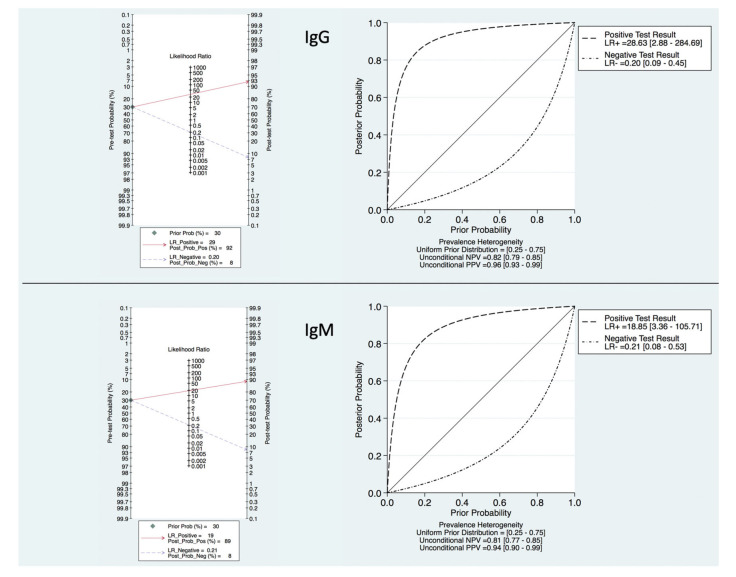
Fagan Nomogram and Probability Modifying Plot.

Based upon the prevalence of 50%, the PPV and NPV was determined to be 96% and 77% for IgG and 95% and 82% for IgM, respectively. Analyses of IgG and IgM serologic-based COVID-19 testing with Fagan plots and probability-modifying plots for positive and negative results were also constructed to determine the meaningfulness or clinical utility (Figure 4). With a pre-test probability of 30%, if a patient tests positive for IgG and IgM, the post-test probability that the patient truly has a history of SARS-CoV-2 infection or acute COVID-19 would be approximately 92% and 89%, respectively. Alternatively, if the patient tests negative, the post-test probability that the patient has a history of an acute infection would be approximately 8% for both IgG and IgM serologic-based testing.

## DISCUSSION

Based upon the results of this systematic review and meta-analysis that included 5 studies and over 1650 patients the performance of serologic-based testing should be considered less than ideal, considering the sensitivities of 81% (IgG) and 80% (IgM) and negative predictive values of 77% (IgG) and 82% (IgM).

Despite increase testing worldwide, there continues to be confusion among healthcare professionals and the public about prioritization of testing and interpretation of results. According to the WHO, widespread testing of symptomatic and asymptomatic individuals is critical to controlling spread of the COVID-19 pandemic (16). Nevertheless, it is important to fully understand the characteristics of available diagnostic tests before public policy recommendations can be made based upon their results. The impact of variable COVID-19 prevalence and clinician pre-test probability on specific test perfomance are also important to understand when developing public health policies.

Currently, the standard method for the diagnosis of COVID-19 is testing of viral RNA by molecular methods, usually via RT-PCR. However, this technique has several limitations. Respiratory shedding of virus peaks at the end of the first week after infection, just prior to the development of symptoms or early in the disease course. Additionally, a single negative NAAT-based swab may result in a false negative result requiring a need for repeat testing if clinical suspicion remains high (16,41,42). NAAT testing also requires substantial equipment, reagents, and expertise and is often carried out in large laboratories. Moreover, swabs must be taken correctly and transported in viral transport medium (14,21,43). Therefore, validated rapid tests are urgently needed to provide more timely information for both diagnosis and public health interventions (44-46).

Antibody tests are primarily used to determine if a person has previously been infected with COVID-19. Antibodies may be detected by conventional ELISA testing or with near-patient lateral flow devices. These types of tests can deliver results in a short time from a few drops of blood obtained by finger prick. However, serologic-based tests typically combine tests for IgM and IgG and may not become positive until the second week of infection and sensitivity may be lower after asymptomatic infection. The WHO reccomends paired samples for confirmation with the initial sample collected in the first week of illness and the second ideally collected 2-4 weeks later, only once validated serology tests are available (16). Additionally, cross-reactivity to other coronaviruses can be challenging (47). Yet, despite these disadvantages, widespread availability of serologic-based testing for antibodies may still provide impact on a global scale. Identification of resolved past infection could allow for individuals to return to work, on the assumption that past infection confers some level of immunity (48-51).

In this meta-analysis, the prevalence of COVID-19 was remarkably high (>50%), though the exact burden of disease is currently unclear given the need for increased testing. The unknown prevalence of COVID-19 makes further interpretation of individual study results challenging. Positive and negative predictive values are influence by the prevalence of disease of the population being testing. Given the seemingly high prevalence of COVID-19 in this meta-analysis these results may actually over-estimate the value of serologic-based testing compared to when the testing is performed in a population with a low prevalence.

The rate of false positive results is important to understand as this may lead to unnecessary hospitalization, treatment, or quarantine. Unfortunately, the studies in this meta-analysis are limited in their ability to assess false positives due to the potential for false negatives of the reference standard.

While there are concerns regarding the false positive rate associated with antibody testing, the rate of false negatives during a pandemic is even more worisome. If an infected person receives a false negative IgM result they remain active in the infected state, and may continue to spread the disease. The impact of IgG false negatives in the asymptomatic population is less clear. There is still a risk for asymptomatic spread, however, the effect on presumed immunity conferd by IgG may be less problematic as there are currently no studies that have demonstrated serologic-based IgG confirmation provides real or sustained protection against the virus. It is important to clarify all of these issues if serologic testing is to be used as a roadmap for reopeing the global economy. Overall, given the low sensitivity and specificity of serologic-based testing, this meta-analysis supports the assertion that an unreliable test is worse than no testing at all. It should also be emphasized that a high-quality test is only effective in a carefully designed strategy.

It is important to recognize our study has some limitations. COVID-19 is a novel disease, therefore there remains a scarcity of high-quality studies available in the literature - potentially making these results less generalizable. It should be noted, however, the quality of studies were considered adequate in our analysis, and according to the QUADAS-2, the index test, reference standard, and flow and timing demonstrated low risk of bias. All studies used RT-PCR as a reference standard. However, it is well known that this test has a considerable false-negative rate (16,21,43). Additionally, the significant heterogeneity seen in this meta-analysis is likely reflective of the early course of disease as well as the different testing platforms designed by various companies. Moreover, data regarding the clinical significance and diagnostic validity of each test, such as patient characteristics, symptoms, and time of sample collection after symptom onset , were not provided in some studies. It is already known that specific IgM and IgG antibodies start to become detactable after 4 to 5 days, with positive IgM antibodies in 70% of symptomatic patients by days 8 to 14 and 90% of total antibody positive by days 11 to 24. IgG reactivity is thought to reach more than 90% after several more weeks, but duration of this antibody response is not yet known (22,42,49,52,53). Lastly, this study was analyzed according to the Cochrane Diagnostic Test Accuracy working group methodology. Although limited by existing data (suggesting at least 10 studies be included to perform assessment of publication bias according to the Cochrane Handbook for Systematic Reviews), we made the decision to perform Deeks’ funnel plot asymmetry and believe it remains an accurate representation and summary of all available data.

In conclusion, current literature-based evidence of serologic testing for COVID-19 suggests that this strategy may not be ready to guide future policy decisions and serve as a roadmap for reopening the global economy. The low-to-moderate sensitivity and specificity found in this meta-analysis, will hopefully spark further investigation and innovation for improved testing. Furthermore, real-world diagnostic testing results from high and low prevalence areas are crucial prior to routine implementation. At this time, it is challenging for the authors to draw firm conclusions regarding the absolute utility of serologic-based testing, beyond the simple need to improve diagnostic testing characteristics. Strategies to minimize the amount of false negative testing are critical. Based upon the results of this meta-analysis, we propose further investment in novel testing strategies and further investigation to better clarify test characteristics in various populations, prior to promoting public health policies that specifically rely on these diagnostic tests.

## AUTHOR CONTRIBUTIONS

Moura DTH was responsible for the study conception and design, manuscript preparation and critical revisions. McCarty TR and Ribeiro IB were responsible for the acquisition of data, statistical analyses, data interpretation, critical revisions. Funari MP, Oliveira PVAG, Miranda Neto AA, Monte Júnior ES, Tustumi F were responsible for the data acquisition and interpretation, and statistical analyses. Bernardo WM and Moura EGH were responsible for the statistical analyses, data interpretation and critical revisions. Thompson CC was responsible for the study conception and design, and critical revisions. All of the authors approved the final version of the manuscript.

## Figures and Tables

**Table 1:** Characteristics and results of individual studies.

							IgM			IgG		
Author	Design	N	Mean age (years)	Male sex (%)	Time after symptoms onset (days)	Symptoms	Sensitivity	Specificity	Accuracy	Sensitivity	Specificity	Accuracy
Xiang et al. (37)	Case-control	150	50	33	3 to 40	Fever, respiratory symptoms and pneumonia	0.83	0.95	0.88	0.77	1.00	0.88
Yunbao et al. (38)	Cross-sectional	105	58	46	0 to 34	Pneumonia	0.56	0.64	0.52	0.55	0.41	0.52
Zhong et al. 39	Cross-sectional	47	48	34	Mean: 14.6	Not reported	0.98	1.00	0.99	0.98	1.00	0.99
Jin et al. (40)	Case-control	60	47	40	Median: 18	Fever, cough, fatigue and sputum	0.48	1.00	0.90	0.89	0.91	0.90
Li et al. (22)	Cross-sectional	525	Not reported	Not reported	Not reported	Not reported	0.83	0.91	0.77	0.71	0.98	0.77

**Supplementary File 1:**
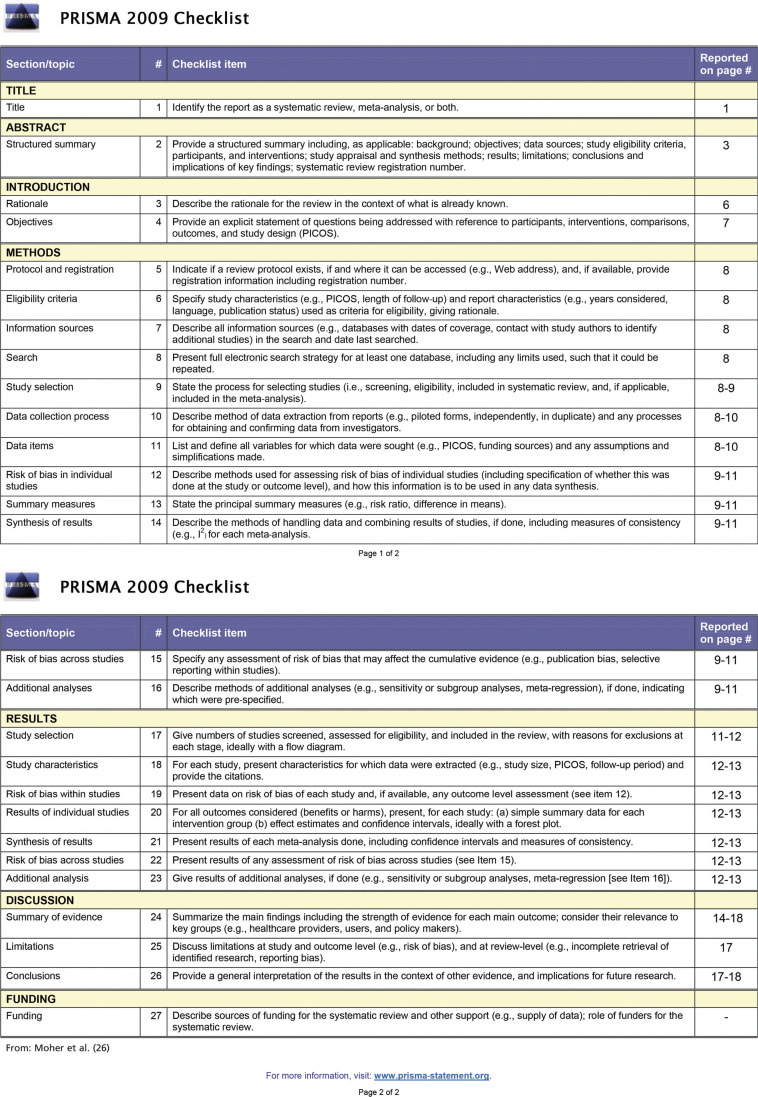
PRISMA 2009 Checklist.

**Supplementary File 2:**
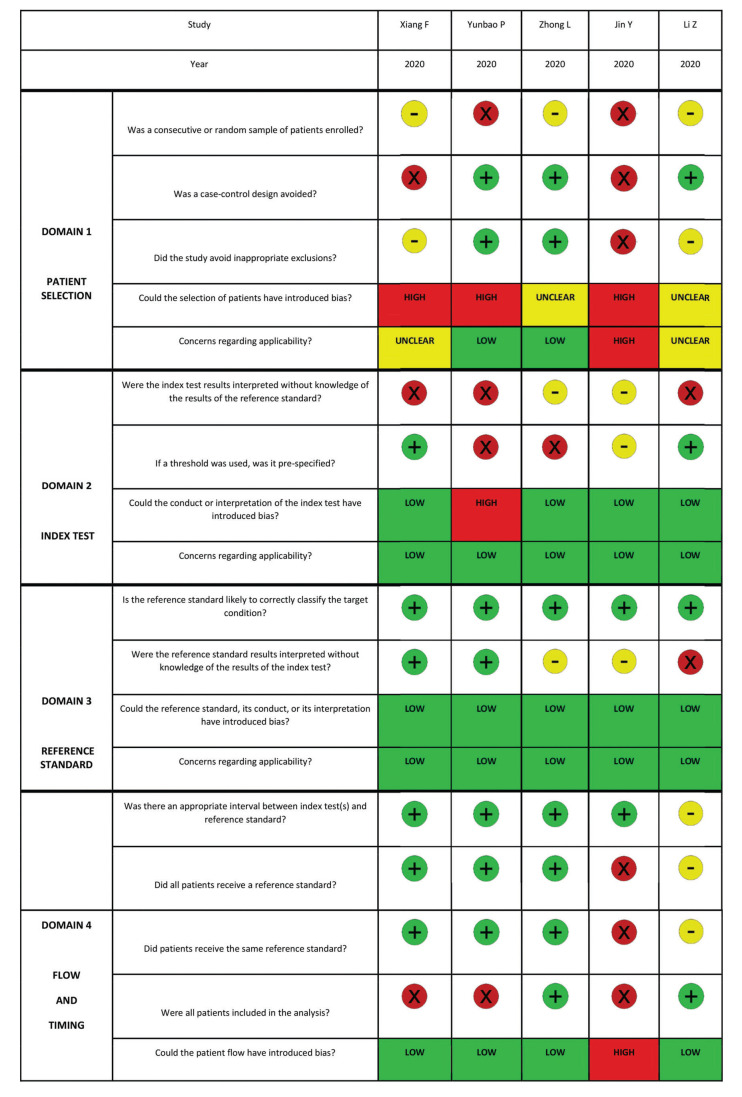
Risk of bias assessment according to QUADAS-2.
